# Bridging clinical care and lived experience: early implementation of longitudinal health-related quality of life monitoring in congenital heart disease

**DOI:** 10.3389/fped.2026.1701283

**Published:** 2026-02-18

**Authors:** Jana Willems, Philipp A. Müller, Anna Erbers, Franziska Renninger, Alina Stricker, Vera König, Rouven Kubicki, Hannah E. Kappler, Alexej Bobrowski, Brigitte Stiller, Christoph Zürn

**Affiliations:** 1 Section of Health Care Research and Rehabilitation Research, Institute of Medical Biometry and Statistics, Medical Faculty and Medical Center - University of Freiburg, Freiburg, Germany; 2 Department of Congenital Heart Defects and Pediatric Cardiology, University Heart Center Freiburg, Medical Center-University of Freiburg, Faculty of Medicine, University of Freiburg, Freiburg, Germany

**Keywords:** health reporting system, health-related quality of life, longitudinal quality of life data, patient centered care, patient reported outcome measure, targeted intervention

## Abstract

**Background:**

Congenital heart defects (CHD) are the most common congenital malformations, and, although survival rates now exceed 90%, children remain at risk for long-term psychomotor, cognitive, and psychosocial difficulties. Routine integration of patient-reported outcome measures (PROMs) into pediatric cardiology offers the potential to detect vulnerabilities early, guide preventive interventions, and facilitate patient- and family-centered care. However, practical implementation in routine settings remains challenging.

**Objectives:**

This pilot study describes the early feasibility and implementation experience, and process adaptations for embedding the Pediatric Quality of Life Inventory™ Cardiac Module (PedsQL CM) into routine pediatric cardiology care, with a focus on the development of a pragmatic implementation model, aiming to enable longitudinal health-related quality of life (HRQoL) monitoring in children with CHD following surgical or catheter-based interventions.

**Methods:**

We are conducting a prospective, single-center, non-interventional study at the University Medical Center Freiburg, enrolling children aged 2–17 years undergoing cardiac surgery or catheterization. The PedsQL CM, available in eight languages in age-appropriate self- and parent-proxy versions, is administered at baseline (hospital admission) and at 3, 6, 9, and 12 months post-intervention. Surveys are implemented in REDCap and distributed via QR codes and automated invitations. Clinical data (diagnosis, intervention details, perioperative parameters) are extracted from the institutional research database for linkage with HRQoL trajectories. Implementation process data are collected systematically, capturing feasibility, acceptability, barriers, and facilitators.

**Preliminary results:**

By mid-study, 77 families have been enrolled. Key facilitators include the visible study team presence, iterative adaptations (e.g., a user-friendly guidance document, filter questions), automation, and interdisciplinary psychosocial team involvement. Barriers comprise limited staff resources, partial IT integration, and variable clinician engagement. Early HRQoL data reflect evidence-based patterns, with generally high scores in younger children and a downward trend during adolescence.

**Conclusion:**

Embedding systematic, longitudinal HRQoL assessment in pediatric cardiology is feasible but requires sustained institutional commitment, robust workflows, and interdisciplinary collaboration. Disease-specific PROMs such as the PedsQL CM provide clinically meaningful insights that support proactive, individualized care. Multicenter collaboration and the extension of PROM pathways across pediatric subspecialties will be key to addressing coverage gaps, optimizing lifelong outcomes, and establishing best practice frameworks to integrate PROMs.

## 1 Introduction

Congenital heart defects (CHD) are the most common congenital organ malformations, affecting approximately 1% of all live births in Germany (1). Of these, an estimated 20%–25% require cardiac surgery or diagnostic and interventional catheter procedures during childhood or adolescence (2). Advances in medical and surgical care have led to a survival rate exceeding 90% for children with CHD, thereby necessitating lifelong, specialized follow-up. As survival rates have risen, the clinical and scientific focus has shifted from mere survival to long-term outcomes, including psychomotor, cognitive, linguistic, and psychosocial sequelae (3, 4). Children with more complex CHD carry a particular risk for developmental and psychosocial impairments. However, the therapeutic success in CHD management is still primarily measured in terms of survival, event-free intervals, and physical performance. While these parameters are essential for quality control, they fail to fully capture patient- and family-centered outcomes. Interprofessional follow-up and support programs are critical—not only for clinical management but also for targeting preventive interventions and optimizing patient and family quality of life (5). Meeting these needs requires robust, systematic data on health-related quality of life (HRQoL) in pediatric CHD.

Patient-reported outcome measures (PROMs) are indispensable for assessing HRQoL and other patient-centered endpoints in clinical contexts (6). Routine use of PROMs in pediatric cardiology is known to improve communication, facilitate the early detection of hidden morbidities, and support personalized care (7). HRQoL instruments such as the Pediatric Quality of Life Inventory™ (PedsQL) have proven feasible and clinically beneficial. Uzark et al. revealed that clinicians, using PedsQL, identified substantial problems in 38% of patients with known heart disease, resulting in targeted interventions in 30% while considering the tool easy, efficient, and informative (7). Similarly, multicenter validation of the Pediatric Cardiac Quality of Life Inventory (PCQLI) in over 1:600 patient-parent pairs demonstrated associations between lower HRQoL scores and increasing cardiac complexity, surgical burden, and healthcare usage (8).

This longitudinal pilot study documents the integration of the PedsQL Cardiac Module into pediatric cardiology routine care at a German university hospital, presenting methodology and implementation strategies to enhance patient-centered HRQoL assessment relying on the most recent evidence and practice guidelines (6, 9). Accordingly, the primary contribution of this article lies in transparently reporting the implementation process, required infrastructure, and practical adaptations under real-world clinical conditions, rather than in evaluating clinical outcomes or intervention effects.

Throughout this manuscript, the term “PROM” refers specifically to standardized patient-reported outcome measures assessing health-related quality of life (HRQoL), and “implementation” denotes the structured integration of these measures into routine clinical workflows.

## 2 Materials and methods

### 2.1 Study objective

This prospective, single-center, non-interventional longitudinal study aims to systematically assess HRQoL via Pediatric Quality of Life Inventory™ Cardiac Module (PedsQL CM) among children with CHD admitted for either cardiac surgery or cardiac catheterization at the Department of Congenital Heart Disease, University Medical Center Freiburg (DRKS-ID: DRKS00028565).

Baseline assessment is performed at hospital admission, with follow-up at three, six, nine, and 12 months post-intervention. Families have been enrolled consecutively since October 2024 and will continue to be enrolled likewise through October 2025; the follow-up period lasts until October 2026.

Our primary objective is to track the longitudinal evolution of HRQoL post-intervention, while the secondary objective is to identify individual physical, emotional, social, and school-related needs from the perspectives of patients and families. Questionnaire responses are systematically linked with relevant clinical data (see section 2.3).

### 2.2 Pediatric quality of life inventory™ (cardiac module)

The PedsQL is grounded in the WHO's definition of quality of life and assesses HRQoL in both healthy and chronically ill children (10). The core questionnaire covers physical, emotional, social, and school functioning.

The disease-specific Cardiac Module was developed for children with CHD and is available as:

Self-report from ages 8–18;
Parent-proxy report from ages 2–18;
An age-adapted self-report (5–7 years) that must be interviewer-administered but which was not feasible in this study due to limited personnel resources.

Table 1 illustrates the PedsQL CM age ranges and respondent types in this study. We use the PedsQL CM in eight languages (German, English, French, Spanish, Portuguese, Italian, Turkish, Russian).

**Table 1:** Administered PedsQL CM age ranges and respondent types.

Age	Proxy-report	Self-report
2–4 yrs.	X	
5–7 yrs.	X	
8–12 yrs.	X	X
13–18 yrs.	X	X

The Cardiac Module covers 23–27 items (age- and medication-dependent) across six domains:

Heart problems and treatment difficulties
Treatment issues II: Difficulties with cardiac medication
Perceived physical appearance problems
Anxiety related to treatment
Cognitive problems
Communication problems

Responses use a five-point Likert scale (0 = never; 4 = almost always); raw scores are transformed to a 0–100 scale, with higher values reflecting fewer problems/better HRQoL.

### 2.3 Collection of clinical parameters

In addition to the questionnaire data, we extract a comprehensive set of clinical parameters from the Freiburg Database for Research in Cardiology (FreDariC) covering:

Age at intervention
Sex
Weight
Type of intervention
Diagnosis and procedure
Various perioperative markers (e.g., The Society of Thoracic Surgeons-European Association for Cardio-Thoracic Surgery (STAT) mortality score, duration of procedure and cardiopulmonary bypass, delayed sternal closure, duration of invasive ventilation, length of stay in pediatric intensive care ward/hospital and follow-up frequency as well as potential death to cancel follow-up surveys)

Data linkage for analysis relies on unique patient identifiers.

### 2.4 Patient sample and recruitment

Recruitment began in October 2024. Children aged 2–17 years scheduled for cardiac surgery or catheterization are eligible. Although the PedsQL Cardiac Module is validated for individuals up to 18 years of age, eligibility in this study is restricted to those <18, reflecting the upper age limit for pediatric cardiology care at our institution. Written informed consent is obtained after briefing by the staff or research team, and the recruitment target is *n* = 150 by the end of 2025. If a child dies during the data collection period, all further follow-up invitations are canceled and the family is removed from the study (exclusion criterion). The protocol has been approved by our local ethics committee (Reference No. 22-1451-S1).

### 2.5 Study procedures and data collection

We are administering the PedsQL CM as an online survey using the secure web-based platform REDCap (Research Electronic Data Capture, version 14.5.43, Vanderbilt University), accessed through QR codes at admission. Eligible patients are screened twice weekly during inpatient admissions. Baseline surveys are completed on-site (using provided tablets or the family's own mobile device), typically with support from the research team or staff. Posters are displayed throughout the ward with QR codes to facilitate access. Follow-up links are distributed automatically via REDCap at scheduled intervals after discharge (3, 6, 9, 12 months). We provide regular reminders for participation at scheduled (outpatient) follow-up visits to encourage completion.

### 2.6 Data management and analysis

Pseudonymized study data are stored securely on internal hospital servers. Data analyses are primarily descriptive and performed using SPSS (IBM SPSS Statistics, version 29). Our results will be stratified by clinical parameters (e.g., underlying CHD), allowing subgroup analyses. Longitudinal HRQoL outcomes will be interpreted in the context of clinical trajectories, with particular attention to CHD type and complexity. Incomplete questionnaires (less than 50% of subscale items answered; according to the PedsQL scoring manual) are excluded. REDCap's logic minimizes missing items; medication items are bypassed if not applicable.

Once full data collection is completed, planned analyses will extend to exploratory inferential and longitudinal approaches, including group comparisons across disease complexity strata, multivariable regression models to examine clinical determinants of HRQoL domains, and longitudinal trajectory analyses across repeated measurement points. These analyses will be explicitly framed as exploratory and hypothesis-generating.

### 2.7 Implementation process documentation

Although we are not relying on a formal implementation science framework such as systematic process evaluation, we are ensuring pragmatic, structured documentation to capture relevant aspects of the PedsQL CM's integration. Process observations and feedback from clinical staff, participating families, and study personnel are recorded throughout the study period. Feedback is routinely reviewed during regular team meetings and used for iterative optimization. The main domains are:

Feasibility: Practical challenges and solutions during patient recruitment, survey distribution (e.g., technical issues, time requirements), and completion rates (baseline and follow-up).
Acceptability: Informal feedback from families regarding questionnaire clarity, digital accessibility, perceived relevance, and support offered during completion.
Barriers and Facilitators: Emerging barriers and effective facilitators are continuously noted and addressed.

We document reflections and procedural adaptations to complement the study’s quantitative findings by providing practical insights over time and to support quality improvement. Wherever possible, adaptations have been guided by expert recommendations for PROM implementation (e.g., automated workflows, minimized burden, and robust support). Preliminary implementation process findings will be reported transparently and discussed below in section 3.

## 3 Preliminary results

This prospective, longitudinal pilot study marks an essential initial step toward integrating routine, longitudinal HRQoL assessment within pediatric cardiology care in Germany. It is our response to the growing consensus that clinical success in pediatric CHD management needs to be expanded by assessing this diverse patient population's lived experiences to identify risk areas early, inform preventive interventions, and ultimately foster improvements in HRQoL.

### 3.1 Implementation process and early observations

Since enrolling 77 families in the early implementation phase, we have identified the main facilitators and persistent barriers presented below.

#### 3.1.1 Facilitators

##### 3.1.1.1 Low-threshold integration

PROM integration is adopted at a low threshold (i.e., with minimal procedural or institutional barriers or prerequisites, allowing easy incorporation into everyday clinical workflows). However, it requires the study team's strong presence and visibility to ensure that our assessment is communicated formally during routine care processes. The study team is consistently on hand to address questions, provide support, and actively follow up with families and clinical staff to monitor progress and emerging challenges. Ongoing, hands-on support, brief and practical staff training, active involvement of on-site doctoral candidates as well as visible recruitment materials (QR posters) increase engagement and streamline recruitment. The presentation of interim results to nursing staff serves to deepen understanding of our study objectives, promoting sustained support throughout the implementation process and strengthening the legitimacy of PROM management within the clinical workflow.

##### 3.1.1.2 Iterative adaptation and participant support

To promote high acceptance and data quality, we engage participants actively through personal reminders and troubleshooting support during questionnaire completion. Early dropouts (often due to inadequate staff support because of resource constraints) led us to introduce a concise, user-friendly guidance document (so-called “cheat sheet”) to help families complete the survey independently and reduce on-site study team time to a realistically manageable degree. Additionally, after receiving family feedback that some items in the 2–4-year age group could not be reliably answered (e.g., with nonverbal children), we implemented a filter question in line with the PedsQL manual to identify insufficient response cases early. This pragmatic adaptation helps minimize unnecessary burden (for families and study team), reduces early drop-outs, and improves overall data completeness and validity.

##### 3.1.1.3 Automated systems

REDCap's automated invitations enable robust, scheduled follow-up in the appropriate language, while dynamically recording participant's age range at each time to indicate that the appropriate age-specific PedsQL CM version was administered. Integrating the PROM status (“PedsQL CM completed?”) in the clinical information system serves as a structured prompt for clinical staff and formalizes the PROM as an element of patient care.

##### 3.1.1.4 Use of a disease-specific PROM

While many studies rely on generic instruments, the application of a disease-specific PROM enables a much more nuanced and clinically meaningful HRQoL assessment. By employing the PedsQL CM, this pilot study achieves greater specificity and depth in capturing the lived experiences of a CHD patient population, thereby enhancing the practical relevance and potential impact of results in the context of routine clinical care.

##### 3.1.1.5 Multi-language administration

The use of the PedsQL CM in eight languages supports the inclusion of families with varying language backgrounds, broadening the reach and representativeness of our findings.

##### 3.1.1.6 Interdisciplinary team involvement

The collection of PROM data introduces additional responsibilities, particularly in identifying and addressing psychological, social, and informational needs. To effectively respond to these, we established regular interdisciplinary case conferences involving our comprehensive psychosocial team that includes psychologists, social workers, and pastoral care providers. We are in the process of collaboratively developing targeted support strategies informed by PedsQL CM data.

#### 3.1.2 Barriers

##### 3.1.2.1 Resource constraints

Resource constraints hinder routine implementation, particularly among the clinical (nursing) staff who are most involved in recruitment. The high demands and fast pace of day-to-day clinical operations (e.g., frequent staff turnover, unpredictable workloads, and competing priorities) often leave limited time for additional tasks such as PROM administration. These pressures can lead to the deprioritization of non-mandatory elements, variability in adherence, and missed opportunities for consistent patient engagement. This highlights the necessity of a dedicated “PROM team”.

##### 3.1.2.2 Timing of study inclusion

The timing of study inclusion within routine clinical care is critically important. Planned admissions typically occur the day before surgery or cardiac catheterization, implying that required diagnostic procedures cannot be postponed on the day of hospitalization. Under these conditions, PROM implementation may be deprioritized or overlooked. Additionally, the stress experienced by families prior to the intervention may reduce willingness to participate and impact both the content of responses and completion rates.

##### 3.1.2.3 Clinical workflow and technical issues

Technical issues comprised automated follow-up-emails being filtered as spam and a lack of broad IT integration. Due to the lack of standardized integration into clinical workflows, HRQoL data could not be used in real time to inform clinical consultations or related decision-making processes. Although we added the PROM status to the clinical information system, this integration occurred as a “study team initiative” rather than through a formal, institution-wide directive. As a result, consistent adherence relies largely on individual staff engagement rather than systematic enforcement.

##### 3.1.2.4 Cultural hurdles remain

Some healthcare providers perceive HRQoL metrics as less relevant than clinical outcomes, resulting in hesitant or inconsistent PROM administration.

These issues underscore that PROM integration is an ongoing change management process, requiring both technical and operational solutions and sustained institutional and cultural commitment as well as systematic stakeholder engagement in patient-centered care.

## 4 Discussion

### 4.1 Towards sustainable, patient/family-centered implementation

Our ongoing experience suggests the following prerequisites for successful, widespread PROM integration in alignment with evidence from pediatric implementation science.

### 4.2 Organizational preconditions

Organizational commitment, systematic training, and robust clinical pathways are fundamental for scalable, sustainable PROM integration (11, 12). Rather than relying on “ad hoc, initiative-driven onboarding”, successful implementation requires institution-wide education protocols and clear procedural standards. Evidence consistently shows that sustainable PROM integration depends on coordinated organizational strategies, including defined roles, sufficient resources, structured training, and feedback mechanisms (11, 12). Common barriers such as limited awareness, perceived low utility, and resource constraints can only be overcome through coordinated education efforts, adaptable workflows, and sustained institutional support. Dedicated implementation teams with protected time are a recurring prerequisite across settings. At our center, the next milestones will be to establish unified PROM pathways across all pediatric subspecialties, i.e., within the Social Pediatric Center.

### 4.3 Infrastructure and digital integration

Real-time user-friendly technical infrastructure and digital clinical integration enables the immediate discussion of PROM results during consultations and supports tailored intervention planning (13, 14). However, such advances require standardized workflows, reliable IT support, and clear procedures for data capture and feedback to ensure consistent use across clinical settings.

### 4.4 Automated clinical response systems and clear workflows

Automated alert and response systems represent a promising advancement in comprehensive and responsive care. These systems enable immediate escalation to ancillary services (e.g., psychology, social work, pastoral care, or community family networks) when problematic scores are identified, creating responsive safety nets. The integration of such systems requires careful consideration of clinical workflow design and staff training. The evidence suggests that successful real-time PROM implementation depends on providing clinicians with easily interpretable visualizations, automated documentation support, and clear workflows for responding to identified concerns (13).

### 4.5 Stakeholder engagement

Active engagement with families and multidisciplinary care teams should be strengthened to ensure that PROM selection, data interpretation, and follow-up interventions are meaningful and doable. Van der Wees et al. emphasized the importance of engaging all stakeholders, including children, adolescents, and caregivers, in the selection of appropriate PROMs and development of specific protocols for assessing timing, administration methods, and clinical pathways (15). There is ample evidence that patient and family involvement in implementation planning is crucial for selecting measures that reflect their priorities and designing workflows that promote engagement (11). Initiatives such as the Patient Advisory Board at the University Medical Center Freiburg exemplify how co-design can support culturally appropriate, patient-centered assessment approaches (16).

### 4.6 Continuous quality improvement and implementation evaluation

Continuous quality improvement through systematic process evaluation using established frameworks of implementation science (e.g., RE-AIM; a framework to guide the planning and evaluation of programs according to outcomes: Reach, Effectiveness, Adoption, Implementation, and Maintenance (17)) should be embedded as a core component of any PROM program to understand contextual factors influencing implementation success, identify replicable intervention components, and develop evidence-based strategies for overcoming common barriers (11, 12). This includes regular monitoring of completion rates, acceptability, and perceived clinical utility, combined with iterative adaptation based on stakeholder feedback. Successful programs implement phased rollout approaches with comprehensive stakeholder engagement, dedicated implementation teams, and the systematic use of “change champions” to support adoption (12). Quality improvement initiatives should regularly evaluate the impact of PROMs on clinical care processes and identify mechanisms to optimize their use for improving overall care delivery (9).

### 4.7 Proactive, targeted interventions enabled by longitudinal HRQoL monitoring

Longitudinal research demonstrates that HRQoL in children and adolescents with CHD is dynamic, varying across developmental stages, patient subgroups, and clinical complexity (7, 18). While younger children often report scores comparable to or exceeding those of healthy peers (19, 20), several studies have revealed a tendency toward a decline with increasing age (particularly during adolescence) - most pronounced in specific subgroups, e.g., those with an elevated body mass index. Importantly, the vulnerability to worsening HRQoL, including a higher risk for anxiety and depression, becomes especially conspicuous during transitional phases. These phases coincide with greater disease awareness, emerging physical limitations (including new comorbidities), and growing psychosocial challenges, such as body image concerns and social integration issues (4, 18, 21).

Given the unpredictable and individual nature of HRQoL trajectories in pediatric CHD, it is essential to routinely integrate longitudinal HRQoL assessment within standard care pathways (7, 20, 21).

In our cohort, where nearly half of all children undergo cardiac surgery in infancy, systematic, risk- and age-stratified HRQoL monitoring is especially compelling and advisable. Our early interim data reflect patterns reported in the literature, indicating a downward HRQoL trajectory with age, although final analyses are pending. Tracking individual patient pathways enables care teams to identify high-risk periods and subgroups, guiding proactive and tailored interventions. This approach transforms PROMs from research instruments into practical clinical decision tools, facilitating the timely activation of psychosocial and rehabilitative resources, strengthening cross-disciplinary collaboration, and ultimately supporting optimal health outcomes and developmental trajectories from childhood through adolescence (4).

### 4.8 Limitations and outlook

A major limitation is the PedsQL CM's age coverage gap, as it cannot be administered to (parents of) children under two years. Given that approximately half of the children with CHD in our cohort undergo cardiac surgery within their first year of life, this constitutes a substantial blind spot in capturing the earliest patient experiences. Complementary strategies are needed for this age group.

Furthermore, the gold standard for HRQoL assessment remains direct self-reporting (22). While the PedsQL CM offers an age-adapted self-report for ages 5–7, interviewer-assisted completion was not feasible in this pilot study, potentially limiting the data quality for younger participants. Child self- and parent proxy-reports have revealed only moderate concordance in certain HRQoL domains (especially emotional and social well-being), calling for better proxy tools and systematic assessments of self/proxy-report agreement across pediatric age groups (23).

The PedsQL CM is robust in capturing child functioning, but it captures neither the full spectrum of HRQoL nor deeper family impact resulting from CHD. Incorporating additional tools, like the PedsQL Family Impact Module or the Family Impact Scale/Familien-Belastungs-Fragebogen (24), would enhance our understanding of children's lived experience. Additional modules assessing healthcare satisfaction (e.g., the PedsQL Healthcare Satisfaction Module) could enhance coverage as well.

Not having a healthy control group and the restricted tracking of confounding variables (e.g., puberty, mental health comorbidities, socio-economic status, and healthcare access disparities) are additional methodological limitations. Our one-year-follow-up period restricts insights into long-term trajectories, underscoring the necessity for extended observation periods and integration of HRQoL assessment within routine care. The absence of parental HRQoL assessment—despite clear evidence linking parent and child quality of life, and demonstrating the impact of parental well-being on patient outcomes—represents an additional limitation (25). Targeted interventions addressing parental needs, especially in families with very young children, merit incorporation into future models of care. Furthermore, incorporating qualitative approaches such as patient and family interviews would provide valuable context for interpreting PROM data, illuminating the relationship between measured scores and reported lived experiences.

Like many pilot studies, organizational resource constraints, evolving infrastructure, and the lack of formalized implementation protocols limit data consistency and generalizability. Nevertheless, these initial steps establish an important foundation for iterative, quality-driven development. Our primary focus remains facilitating individualized, score-based interventions as advocated by Uzark et al. (7)

## 5 Conclusion

This pilot study demonstrates the feasibility and early utility of embedding systematic longitudinal HRQoL assessment via the PedsQL Cardiac Module in pediatric cardiology care. Our structured process documentation and iterative optimization revealed key facilitators, including interdisciplinary support, automation, and contextual PROM adaptation, as well as persistent barriers, notably resource limitations and the need for formalized protocols. The sharing of implementation experiences strengthens the growing international evidence supporting evidence-based PROM integration strategies.

The longitudinal, risk-stratified HRQoL monitoring of children who have undergone cardiac surgery is essential for detecting their periods of vulnerability, informing timely, targeted interventions, and ultimately advancing patient- and family-centered care for children with CHD. Addressing current coverage gaps and promoting comprehensive, multicenter approaches are pivotal for optimizing lifelong outcomes and establishing best practices in PROM integration across pediatric specialties; it is the clinical heterogeneity of CHD in particular that underscores the need for collaborative, multicenter research to inform broader implementation efforts and support the ongoing advancement of PROM utilization.

## Data Availability

The original contributions presented in the study are included in the article/Supplementary Material, further inquiries can be directed to the corresponding author.
